# Frequency of *vacA*, *cagA *and *babA2 *virulence markers in *Helicobacter pylori *strains isolated from Mexican patients with chronic gastritis

**DOI:** 10.1186/1476-0711-8-14

**Published:** 2009-04-30

**Authors:** Gloria Luz Paniagua, Eric Monroy, Raymundo Rodríguez, Salvador Arroniz, Cristina Rodríguez, José Luis Cortés, Ausencio Camacho, Erasmo Negrete, Sergio Vaca

**Affiliations:** 1 Facultad de Estudios Superiores Iztacala, Universidad Nacional Autónoma de Mexico, Avenida de los Barrios 1, Los Reyes Iztacala, Tlalnepantla, 54090, Estado de Mexico, Mexico; 2 Hospital General Regional 72 del Instituto Mexicano del Seguro Social, Av. G. Baz s/n, Tlanepantla, 54000, Estado de Mexico, Mexico

## Abstract

**Background:**

*Helicobacter pylori *has been strongly associated with chronic gastritis, peptic and duodenal ulcers, and it is a risk factor for gastric cancer. Three major virulence factors of *H. pylori *have been described: the vacuolating toxin (VacA), the cytotoxin-associated gene product (CagA) and the adhesion protein BabA2. Since considerable geographic diversity in the prevalence of *H. pylori *virulence factors has been reported, the aim of this work was to establish the *H. pylori *and *vacA*, *cagA *and *babA2 *gene status in 238 adult patients, from a marginal urban area of Mexico, with chronic gastritis.

**Methods:**

*H. pylori *was identified in cultures of gastric biopsies by nested PCR. *vacA *and *cagA *genes were detected by multiplex PCR, whereas babA2 gene was identified by conventional PCR.

**Results:**

*H. pylori*-positive biopsies were 143 (60.1%). All *H. pylori *strains were *vacA*^+^; 39.2% were *cagA*^+^; 13.3% were *cagA*^+ ^*babA2*^+ ^and 8.4% were *babA2*^+^. Mexican strains examined possessed the *vacA s1, m1 *(43.4%), *s1, m2 *(24.5%), *s2, m1 *(20.3%) and *s2, m2 *(11.9%) genotypes.

**Conclusion:**

These results show that the Mexican patients suffering chronic gastritis we have studied had a high incidence of infection by *H. pylori*. Forty four percent (63/143) of the *H. pylori *strains analyzed in this work may be considered as highly virulent since they possessed two or three of the virulence markers analyzed: *vacA s1 cagA babA2 *(9.8%, 14/143), *vacA s1 babA2 *(4.9%, 7/143), and *vacA s1 cagA *(29.4%, 42/143). However, a statistically significant correlation was not observed between *vacAs1*, *cagA *and *babA2 *virulence markers (χ^2 ^test; P > 0.05).

## Background

*Helicobacter pylori *is a spiral-shaped Gram-negative bacterium that has been strongly associated with chronic gastritis and peptic ulcer disease [1,2], and it is a risk factor for gastric cancer [3-5]

Three major virulence factors of *H. pylori *have been described: the cytotoxin-associated gene product (CagA), the vacuolating toxin (VacA) and the adhesion protein BabA2. The cytotoxin-associated gene A (CagA) is a protein with a molecular mass of approximately 125–140 kDa, encoded by the *cagA *gene, [6,7], that is translocated into gastric epithelial cells by a type IV secretion system, encoded by the *cag *pathogenicity island (*cag *PAI) [8]. Inside epithelial cells CagA is phosphorylated on tyrosine residues by host cell Src kinases and stimulates cell-signaling pathways [9], which in turn causes elongation of the cell [10] and activation of proto-oncogenes [11].

The vacuolating cytotoxin gene *vacA *is polymorphic, varying in the signal and middle regions. The main signal region alleles are *s1 *and *s2*, whereas the middle region alleles are *m1 *and *m2 *[12,13]. VacA is a toxin that binds to several epithelial receptors [14-16] and forms hexameric pores [17], which later are endocytosed and converted in large vacuoles [18].

The BabA adhesin of *H. pylori *is an outer membrane protein that binds to the fucosylated histo-blood group antigens on the surface of gastric epithelial cells [19,20]. It has been reported that *H. pylori *strains possessing *babA2 *gene, which encodes active BabA adhesin, are associated with increased gastric inflammation [21] and increased risk for duodenal ulcer and adenocarcinoma [22].

*H. pylori *virulence factors frequency varies widely. For instance, *vacAs1 *prevalence fluctuates from 48% [23] to 100% [24] whereas *cagA *prevalence fluctuates from 66.9% [23] to 83.6% [25] and 100% [26]. *babA2 *reported frequencies vary from 46% [27] to 82.3% [28] in South-American countries. Since considerable geographic diversity in the prevalence of *H. pylori *virulence factors has been reported, the aim of this work was to establish the *H. pylori *and *vacA*, *cagA *and *babA2 *gene status in 238 adult patients, from a marginal urban area of Mexico, with chronic gastritis.

## Materials and methods

### Subjects and clinical samples

Two hundred and thirty eight patients, endoscopic diagnosed with chronic gastritis (154 women and 84 men) with an average age of 52.24 years (range 16 to 83), who had undergone endoscopy in Hospital General Regional 72 of the Instituto Mexicano del Seguro Social at Tlalnepantla, Estado de Mexico, Mexico, were included in this study. Written informed consent for participation was obtained from every patient before the study. The ethics committee at Hospital General Regional 72 approved the study protocol in advance. Antral biopsy specimens were evaluated for the presence of *H. pylori *by culture. The genotype profiles of *H. pylori *isolates were determined by PCR.

### *H. pylori *culture

For bacterial culture, biopsy specimens were macerated and homogenized in Brucella Broth and a 100 μL aliquot was inoculated on Casman Agar (Difco) containing 5% horse blood and *H. pylori *selective supplement (Oxoid-SR 147E). Agar plates were incubated in 6% CO_2_, for up to four days. Colonies were identified as *H. pylori *according to standard criteria including negative Gram staining, typical cell morphology, and positive reactions to catalase, oxidase, and urease.

### Identification of *H. pylori *by nested PCR

*H. pylori *DNA was extracted from colonies collected in microcentrifuge tubes containing 125 μL of sterile phosphate-buffered saline. Suspensions were vortexed vigorously for 2 min; the tubes were boiled in a water bath for 15 min, cooled in ice, and centrifuged at 13000 × g for 1 min. DNA in supernatant was stored at -20°C until used as template in PCR.

*H. pylori *was detected by nested PCR. First PCR run was done as described by Li et al., [29] with primers EHC-U (5'-CCCTCACGCCATCAGTCCCAAAAA-3') and EHC-L (5'-AAGAAGTCAAAAACGCCCCAAAAC-3'). Amplification was performed in 25 μL reaction volume containing 1 μL (25 pmol) of each primer (EHC-U and EHC-L, Sigma-Genosys), 2.5 μL 10× Buffer Solution, 17.5 μL nuclease-free water, 3 μL template DNA, 1.5 mmol MgCl_2_, 0.5 U AmpliTaq polimerase and 100 mmol dNTPs (PuRetaqTM Ready-To-GoTM PCR beads). Products were amplified under the following conditions: 5 min at 95°C for initial denaturation followed by 40 cycles of 45 s at 94°C, 45 s at 59°C, and 30 s at 72°C with a final round of 10 min at 72°C in a Corbett Research CGI-96 Thermocycler. A 417 bp product was obtained by this procedure. Second PCR run was done as described by Song et al., [30] with primers ET-5U (5'-GCCAAATCATAAGTCCGCAGAA-3') and ET-5L (5'-TGAGACTTTCCTAGAAGCGGTGTT-3') complementary to an internal fragment of the amplicon obtained with EHC-U and EHC-L primers. Amplification conditions were identical to those of the first run, except that 0.2 μL of the first PCR run product as template, and 25 cycles, were used. A 230 bp amplicon was obtained.

In each experiment, both positive and negative controls, with DNA from H. pylori ATCC 43629 and without template DNA, were included.

### Detection of *cagA*, *vacA*, and *babA2 *by PCR

In order to detect *cagA *and *vacA *alleles, primers and multiplex PCR amplification conditions described by Chattopadhyay, et al., [31] and Atherton et al., [13,32] were used. These PCR protocols detect *cagA *(350 bp amplicon) and distinguish *vacA s1 *(259 bp amplicon) from *vacA s2 *(286 bp amplicon), and *vacA m1 *(567 bp amplicon) from *vacA m2 *(642 bp amplicon). PCR detection of *babA2 *was done as described by Gerhard et al., [22]. PCR products were analyzed by agarose gel electrophoresis at 120 V, 94 mA for 120 min. Gels were stained with ethidium bromide and photographed under UV illumination with Gel Logic 100 system (Kodak).

## Results and discussion

**Figure 1:**
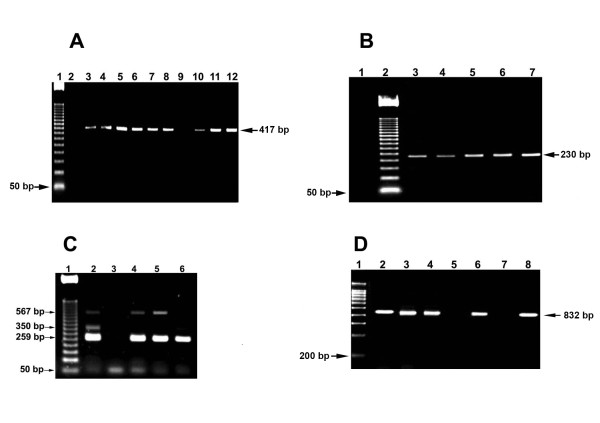
**Identification of *H. pylori *isolated from gastric biopsy samples and genotyping of its main virulence genes by PCR**. Images shown are from representative gel electrophoresis. A: Lane 1, MWM 50 bp-ladder; Lanes 2 and 9 negative control without DNA; Lanes 3–8, 10 and 11, *H. pylori *isolated from gastric biopsy samples (417-bp amplicon); Lane 12, reference strain *H. pylori *ATCC 43629. B: Lane 1, negative control without DNA, Lane 2, MWM 50 bp-ladder; Lanes 3–6, *H. pylori *isolated from gastric biopsy samples (230-bp amplicon); Lane 7, *H. pylori *ATCC 43629. C: Lane 1, MWM 50-bp-ladder; Lane 2, *H. pylori *isolated from gastric biopsy sample (*vacA s1, m1 cagA*); Lane 3, negative control without DNA; Lanes 4–5, *H. pylori *isolated from gastric biopsy samples (*vacA s1, m1*); Lane 6, ATCC 43629. D: Lane 1, MWM 200 bp-ladder; Lane 2, ATCC 43629; Lanes 3,4,6 and 8, *babA2*-positive *H. pylori *isolated from gastric biopsy samples; Lane 5, control without DNA; Lane 7, *babA2*-negative *H. pylory *isolated from gastric biopsy sample.

It is known that more than 50% of the world's human population is colonized by *H. pylori *[33,34]. We report here that *H. pylori *was cultured from 60.1% biopsy samples (143/238) and identified by nested PCR, which rendered the expected 417 bp and 230 bp amplicons (Fig 1A, B) as reported by Li et al., [29] and Song et al., [30]. This result is in agreement with previously reported *H. pylori *prevalence in Mexican people. A community-based national seroprevalence survey of *H. pylori *infection in Mexico showed an overall prevalence of 66%. Twenty-percent of one-year-old children had antibodies against *H. pylori*, with an increased seropositivity of up to 50% in children who were 10 years of age [35]. Variations in prevalence have been reported among particular regions with a prevalence of 86.1% in southeastern Mexico [36] and 47.1% in children from northwestern Mexico [37].

All *H. pylori *strains were positive for the *vacA *gene (Table 1), as evidenced by PCR product sizes, which enabled to differentiate *s *and *m *alleles (Fig. 1C). *s1 m1 *was the most frequent *vacA *allelic combination in the *H. pylori *strains examined, followed by *s1 m2*, *s2 m1 *and *s2 m2 *(Table 1). These results suggest that two thirds of these strains are virulent, as it has been reported that *H. pylori *isolates with *vacAs1m1 *and *vacAs1m2 *allelic combinations exhibit high and low vacuolating activity, respectively, whereas those with *vacAs2 *fails to induce cell vacuolation in vitro [13].

**Table 1:** Frequency of *vacA H. pylori *genotypes in Mexican patients with chronic gastritis

Genotype	Number (%)
*s1m1*	62 (43.4)
*s1m2*	35 (24.5)
*s2m1*	29 (20.3)
*s2m2*	17 (11.9)

	143 (100)

Amplicons of the *cagA *and *babA2 *genes were detected in agarose gels as 350 bp and 832 bp bands, respectively, (Fig. 1C and 1D). Fifty two percent of the *H. pylori *isolates were *cagA-*positive, prevalence less than that reported in other studies from South-American [28,38,39] and Asian countries [40]. In Mexico, prevalence of *H. pylori *infection with CagA-positive strains varies from 47.6% to 63.4% [41]. *cagA*-positive *H. pylori *strains have been associated with the severe mucosa inflammation that underlies peptic ulcer, atrophic gastritis and gastric carcinoma [42-44].

Although *vacAs1 cagA+ H. pylori *strains had been considered as virulent, in a study of *H. pylori *isolates from Mexican patients it was reported that *vacAs1b *and *cagA+ *strains were found at similar frequencies in adults with and without peptic ulcers [45].

*H. pylori *BabA adhesin, encoded by the *babA2 *gene, participates in adhesion of *H. pylori *to Le^b ^antigens on human gastric epithelial cells [19]. The *babA2 *gene was found in only 21.7% of the *H. pylori *isolates (Table 2). This frequency is considerably lesser than *babA2 *reported frequencies, which vary from 46% [27] to 82.3% [28] in South-American countries. It is important to note that PCR detection of *babA2 *in *H. pylori *do not always correlates with its adhesive properties and, conversely, failure to detect *babA2 *by PCR does not mean that the strain is not adherent, as there is substantial allelic variation in *babA2 *gene [46-48].

**Table 2:** Frequency of *cagA *and *babA2 *genes in *H. pylori *isolates

Genotype			Number (%)
*vacA*	*cagA*	*babA2*	

+	+	+	19 (13.3)
+	-	+	12 (8.4)
+	+	-	56 (39.2)
+	-	-	56 (39.2)

			143 (100)

Forty four percent of the *H. pylori *strains analyzed in this work (63/143) possessed two or three of the virulence markers analyzed (Table 3): *vacA s1 cagA babA2 *(9.8%, 14/143), *vacA s1 babA2 *(4.9%, 7/143), and *vacA s1 cagA *(29.4%, 42/143).

**Table 3:** Frequency of *cagA *an *babA2 *genes in *vacAs1*-positive *H. pylori *isolates

Genotype			Number (%)
*vacAs1*	*cagA*	*babA2*	

+	+	+	14 (14.4)
+	-	+	7 (7.2)
+	+	-	42 (43.3)
+	-	-	34 (35.0)

			97 (100)

## Conclusion

Results presented here show that the Mexican patients suffering chronic gastritis we have studied had a high incidence of infection by *H. pylori*, and suggest that 44% of the *H. pylori *strains examined may be considered virulent, since they possessed two or three of the virulence markers analyzed. However, a statistically significant correlation was not observed between *vacAs1*, *cagA *and *babA2 *virulence markers (χ^2 ^test; P > 0.05).

## Competing interests

The authors declare that they have no competing interests.

## Authors' contributions

GP, EM and EN carried out the molecular studies; RR, JC and AC, obtained the gastric biopsy samples; SA and CR carried out the microbiological procedures; SV conceived of the study, and participated in its design and coordination, and drafted the manuscript. All authors read and approved the final manuscript.

## References

[B1] Marshall BJ, Warrren JR (1984). Unidentified curved bacilli in the stomach of patients with gastritis and peptic ulceration. Lancet.

[B2] Petersen WL (1991). *Helicobacter pylori *and peptic ulcer disease. N Engl J Med.

[B3] NIH Consensus Development Panel (1994). *Helicobacter pylori *in peptic ulcer disease. J Am Med Assoc.

[B4] International Agency for Research on Cancer (1994). Schistosomes, liver flukes and *Helicobacter pylori*. IARC Monographs on the Evaluation of Carcinogenic Risks to Humans.

[B5] Isaacson PG (1994). Gastric lymphoma and *Helicobacter pylori*. N Engl J Med.

[B6] Covacci A, Censini S, Bugnoli M, Petracca R, Burroni D, Macchia G, Massone A, Papini E, Xiang Z, Figura N, Rappuoli R (1993). Molecular characterization of the 128-kDa immunodominant antigen of *Helicobacter pylori *associated with cytotoxicity and duodenal ulcer. Proc Natl Acad Sci USA.

[B7] Tummuru MK, Cover TL, Blaser MJ (1993). Cloning and expression of a high-molecular-mass major antigen of *Helicobacter pylori*: evidence of linkage to cytotoxin production. Infect Immun.

[B8] Odenbreit S, Puls J, Sedlmaier B, Gerland E, Fischer W, Hass R (2000). Translocation of *Helicobacter pylori *CagA into gastric epithelial cells by type IV secretion. Science.

[B9] Selbach M, Moese S, Hauck CR, Meyer TF, Backert S (2002). Src is the kinase of the *Helicobacter pylori *CagA protein in vitro and in vivo. J Biol Chem.

[B10] Segal ED, Cha J, Lo J, Falkow S, Tompkins LS (1999). Altered states: involvement of phosphorylated CagA in the induction of host cellular growth changes by *Helicobacter pylori*. Proc Natl Acad Sci USA.

[B11] Meyer-ter-Vehn T, Covacci A, Kist M, Pahl HL (2000). *Helicobacter pylori *activates mitogen-activated protein kinase cascades and induces expression of the proto-oncogenes c-fos and c-jun. J Biol Chem.

[B12] Cover TL, Tummuru MK, Cao P, Thompson SA, Blaser MJ (1994). Divergence of genetic sequences for the vacuolating cytotoxin among *Helicobacter pylori *strains. J Biol Chem.

[B13] Atherton JC, Cao P, Peek RM, Tummuru MK, Blaser MJ, Cover TL (1995). Mosaicism in vacuolating cytotoxin alleles of *Helicobacter pylori*. Association of specific *vacA *types with cytotoxin production and peptic ulceration. J Biol Chem.

[B14] Seto K, Hayashi-Kuwabara Y, Yoneta T, Suda H, Tamaki H (1998). Vacuolation induced by cytotoxin from *Helicobacter pylori *is mediated by EGF receptor in HeLa cells. FEBS Lett.

[B15] Padilla PI, Wada A, Yahiro K, Kimura M, Niidome T, Aoyagi H, Kumatori A, Anami M, Hayashi T, Fujisawa J, Saito H, Moss J, Hirayama T (2000). Morphologic differentiation of HL-60 cells is associated with appearance of RPTP beta and induction of *Helicobacter pylori *VacA sensitivity. J Biol Chem.

[B16] Yahiro K, Wada A, Nakayama M, Kimura T, Ogushi K, Niidome T, Aoyagi H, Yoshino K, Yonezawa K, Moss J, Hirayama T (2003). Protein-tyrosine phosphatase alpha, RPTP alpha, is a *Helicobacter pylori *VacA receptor. J Biol Chem.

[B17] Czajkowsky DM, Iwamoto H, Cover TL, Shao ZF (1999). The vacuolating toxin from *Helicobacter pylori *forms hexameric pores in lipid bilayers at low pH. Proc Natl Acad Sci USA.

[B18] Papini E, de Bernard M, Milia E, Bugnoli M, Zerial M, Rappuoli R, Montecucco C (1994). Cellular vacuoles induced by *Helicobacter pylori *originate from late endosomal compartments. Proc Natl Acad Sci USA.

[B19] Borén T, Falk P, Roth KA, Larson G, Normark S (1993). Attachment of *Helicobacter pylori *to human gastric epithelium mediated by blood group antigens. Science.

[B20] Ilver D, Arnqvist A, Ogren J, Frick IM, Dangeruta K, Incecik ET, Berg DE, Covacci A, Engstrand L, Borén T (1998). *Helicobacter pylori *adhesin binding fucosylated histo-blood group antigens revealed by retagging. Science.

[B21] Prinz C, Schoniger M, Rad R, Becker I, Keiditsch E, Wagenpfeil S, Classen M, Rosch T, Schepp W, Gerhard M (2001). Key importance of the *Helicobacter pylori *adherence factor blood group antigen binding adhesin during chronic gastric inflammation. Cancer Res.

[B22] Gerhard M, Lehn N, Neumayer N, Borén T, Rad R, Schepp W, Miehlke S, Classen M, Prinz C (1999). Clinical relevance of the *Helicobacter pylori *gene for blood-group antigen-binding adhesin. Proc Natl Acad Sci USA.

[B23] Alarcón T, Domingo D, Martinez MJ, López-Brea M (1999). *cagA *gene and *vacA *alleles in Spanish *Helicobacter pylori *clinical isolates from patients of different ages. FEMS Immunol Med Microbiol.

[B24] Chomvarin C, Namwat W, Chaicumpar K, Mairiang P, Sangchan A, Sripa B, Tor-Udom S, Vilaichone RK (2008). Prevalence of *Helicobacter pylori vacA, cagA, iceA *and *babA2 *genotypes in Thai dyspeptic patients. Int J Infect Dis.

[B25] Salehi Z, Jelodar MH, Rassa M, Ahaki M, Mollasalehi H, Mashayekhi F (2009). *Helicobacter pylori cagA *status and peptic ulcer disease in Iran. Dig Dis Sci.

[B26] Pan ZJ, Hulst RW van der, Feller M, Xiao SD, Tytgat GN, Dankert J, van der Ende A (1997). Equally high prevalence of infection with *cagA*-positive *Helicobacter pylori *in Chinese patients with peptic ulcer disease and those with chronic gastritis-associated dyspepsia. J Clin Microbiol.

[B27] Oliveira AG, Santos A, Guerra JB, Rocha GA, Rocha AM Oliveira CA, Cabral MM, Nogueira AM, Queiroz DM (2003). *babA2- *and *cagA*-positive *Helicobacter pylori *strains are associated with duodenal ulcer and gastric carcinoma in Brazil. J Clin Microbiol.

[B28] Torres LE, Melián K, Moreno A, Alonso J, Sabatier CA, Hernández M, Bermúdez L, Rodríguez BL (2009). Prevalence of *vacA, cagA *and *babA2 *genes in Cuban *Helicobacter pylori *isolates. World J Gastroenterol.

[B29] Li C, Musich PR, Ha T, Ferguson DA, Patel NR, Chi DS, Thomas E (1995). High prevalence of *Helicobacter pylori *in saliva demonstrated by a novel PCR assay. J Clin Pathol.

[B30] Song Q, Haller B, Schmid RM, Adler G, Bode G (1999). *Helicobacter pylori *in dental plaque: a comparison of different PCR primer sets. Dig Dis Sci.

[B31] Chattopadhyay S, Patra R, Ramamurthy T, Chowdhury A, Santra A, Dhali GK, Bhattacharya SK, Berg DE, Nair GB, Mukhopadhyay AK (2004). Multiplex PCR assay for rapid detection and genotyping of *Helicobacter pylori *directly from biopsy specimens. J Clin Microbiol.

[B32] Atherton JC, Cover TL, Twells RJ, Morales MR, Hawkey CJ, Blaser MJ (1999). Simple and accurate PCR-based system for typing vacuolating cytotoxin alleles of *Helicobacter pylori*. J Clin Microbiol.

[B33] Parsonnet J (1995). Incidence of *Helicobacter pylori *infection. Aliment Pharmacol Ther.

[B34] Atherton JC (2006). The pathogenesis of *Helicobacter pylori*-induced gastro-duodenal diseases. Annu Rev Pathol Mech Dis.

[B35] Torres J, Leal-Herrera Y, Perez-Perez G, Gomez A, Camorlinga-Ponce M, Cedillo-Rivera R, Tapia-Conyer R, Muñoz O (1998). A community-based seroepidemiological study of *Helicobacter pylori *infection in Mexico. J Infect Dis.

[B36] Gaurner J, Mohar A, Parsonnet J, Halperin D (1993). The association of *Helicobacter pylori *with gastric cancer and preneoplastic gastric lesions in Chiapas, Mexico. Cancer.

[B37] Jiménez-Guerra F, Shetty P, Kurpad A (2000). Prevalence of and risk factors for *Helicobacter pylori *infection in school children in Mexico. Ann Epidemiol.

[B38] Faundez G, Troncoso M, Figueroa G (2002). *cagA *and *vacA *in strains of *Helicobacter pylori *from ulcer and non-ulcerative dyspepsia patients. BMC Gastroenterol.

[B39] Mattar R, dos Santos AF, Eisig JN, Rodrigues TN, Silva FM, Lupinacci RM, Iriya K, Carrilho FJ (2005). No correlation of *babA2 *with *vacA *and *cagA *genotypes of *Helicobacter pylori *and grading of gastritis from peptic ulcer disease patients in Brazil. Helicobacter.

[B40] Maeda S, Ogura K, Yoshida H, Kanai F, Ikenoue T, Kato N, Shiratori Y, Omata M (1998). Major virulence factors, VacA and CagA, are commonly positive in *Helicobacter pylori *isolates in Japan. Gut.

[B41] Torres J, Lopez L, Lazcano E, Camorlinga M, Flores L, Muñoz O (2005). Trends in *Helicobacter pylori *infection and gastric cancer in Mexico. Cancer Epidemiol Biomarkers Prev.

[B42] Kuipers EJ, Pérez-Pérez GI, Meuwissen SG, Blaser MJ (1995). *Helicobacter pylori *and atrophic gastritis: importance of the *cagA *status. J Natl Cancer Inst.

[B43] Blaser MJ, Pérez-Pérez GI, Kleanthous H, Cover TL, Peek RM, Chyou PH, Stemmermann GN, Nomura A (1995). Infection with *Helicobacter pylori *strains possessing *cagA *is associated with an increased risk of developing adenocarcinoma of the stomach. Cancer Res.

[B44] Yamaoka Y, Kodama T, Gutierrez O, Kim JG, Kashima K, Graham DY (1999). Relationship between *Helicobacter pylori iceA, cagA*, and *vacA *status and clinical outcome: studies in four different countries. J Clin Microbiol.

[B45] González-Valencia G, Atherton JC, Muñoz O, Dehesa M, Madrazo-de la Garza A, Torres J (2000). *Helicobacter pylori vacA *and *cagA *genotypes in Mexican adults and children. J Infect Dis.

[B46] Hennig EE, Mernaugh R, Edl J, Cao P, Cover TL (2004). Heterogeneity among *Helicobacter pylori *strains in expression of the outer membrane protein BabA. Infect Immun.

[B47] Olfat FO, Zheng Q, Oleastro M, Voland P, Borén T, Karttunen R, Engstrand L, Rad R, Prinz C, Gerhard M (2005). Correlation of the *Helicobacter pylori *adherence factor BabA with duodenal ulcer disease in four European countries. FEMS Immunol Med Microbiol.

[B48] Colbeck JC, Hansen LM, Fong JM, Solnick JV (2006). Genotyping profile of the outer membrane proteins BabA and BabB in clinical isolates of *Helicobacter pylori*. Infect Immun.

